# Sparse Bayes Tensor and DOA Tracking Inspired Channel Estimation for V2X Millimeter Wave Massive MIMO System

**DOI:** 10.3390/s21124021

**Published:** 2021-06-10

**Authors:** Kaihua Luo, Xiaoping Zhou, Bin Wang, Jifeng Huang, Haichao Liu

**Affiliations:** The College of Information, Mechanical and Electrical Engineering, Shanghai Normal University, Shanghai 200234, China; vc_luokaihua@163.com (K.L.); jfhuang@shnu.edu.cn (J.H.); liuhaichao0609@163.com (H.L.)

**Keywords:** vehicle to everything (V2X), mmWave massive MIMO, channel estimation, direction of arrival (DOA) tracking

## Abstract

Efficient vehicle-to-everything (V2X) communications improve traffic safety, enable autonomous driving, and help to reduce environmental impacts. To achieve these objectives, accurate channel estimation in highly mobile scenarios becomes necessary. However, in the V2X millimeter-wave massive MIMO system, the high mobility of vehicles leads to the rapid time-varying of the wireless channel and results in the existing static channel estimation algorithms no longer applicable. In this paper, we propose a sparse Bayes tensor and DOA tracking inspired channel estimation for V2X millimeter wave massive MIMO system. Specifically, by exploiting the sparse scattering characteristics of the channel, we transform the channel estimation into a sparse recovery problem. In order to reduce the influence of quantization errors, both the receiving and transmitting angle grids should have super-resolution. We obtain the measurement matrix to increase the resolution of the redundant dictionary. Furthermore, we take the low-rank characteristics of the received signals into consideration rather than singly using the traditional sparse prior. Motivated by the sparse Bayes tensor, a direction of arrival (DOA) tracking method is developed to acquire the DOA at the next moment, which equals the sum of the DOA at the previous moment and the offset. The obtained DOA is expected to provide a significant angle information update for tracking fast time-varying vehicular channels. The proposed approach is evaluated over the different speeds of the vehicle scenarios and compared to the other methods. Simulation results validated the theoretical analysis and demonstrate that the proposed solution outperforms a number of state-of-the-art researches.

## 1. Introduction

The last few years have witnessed the advent of the vehicle to everything (V2X) communications deployed in the millimeter-wave (mmWave) band as a means to circumvent the spectrum shortage needed to satisfy the stringent requirements of 5G networks.V2X communications have been initially designed to support active safety and traffic management services. The development of more advanced connected and automated applications requires the exchange of larger amounts of data [1]. For example, automated driving or vehicle formation will require vehicles to exchange raw or processed data of their built-in sensors to improve their perception of the surrounding environment [2,3]. Supporting the data rates and throughput required by this type of advanced applications can hardly be satisfied by sub-6 GHz V2X standards [4,5]. Fortunately, there are abundant underutilized spectrum resources in the millimeter wave band (30 GHz~300 GHz), which can potentially support the required data rate. However, the rain attenuation, severe path loss, and high atmospheric absorption make it difficult to implement these advanced applications. However, massive multi-input multi-output (MIMO) antenna arrays can be packed into small chips at these frequencies and can provide a sufficiently powerful received signal [6,7]. Moreover, massive MIMO antenna arrays help the design of beamforming techniques to direct the signal in a certain direction, which reduces the path loss problem [8]. Therefore, millimeter-wave massive MIMO is a very promising technology that can be used for multi-gigabits-per-second (Gbps) to terabits-per-second (Tbps) wireless links to improve the performance of next-generation V2X applications [9].

To reach the potential of mmWave MIMO systems for V2X communication scenarios, channel state information (CSI) is usually required. However, it is a challenge to estimate the channels in millimeter-wave massive MIMO systems with large-scale antenna arrays and low signal-to-noise ratio (SNR). Several familiar algorithms, such as spectrum estimation [10,11], sparse recovery, and out-of-band information assistance methods, have been well investigated to estimate CSI at a certain time-frequency grid [12,13,14,15,16]. However, a superabundant amount of papers is for a quasi-static channel estimator. Recently, the fast time-varying channel estimation has received more attention [17,18,19]. The authors of [17,18] examined the effects of channel estimation duration and received signal dimension and proposed the application of tensor decomposition theories to estimate CSI. Y. Yang et al. [19] proposed an inter-vehicle cooperation channel estimation (IVC-CE) method and utilizing the position and velocity of a vehicle as a priori information to obtain accurate CSI. The authors of [20] studied proposed an adaptive angle estimation (AAE) algorithm method based on millimeter-wave of the time-variant wireless channel, and transmission frame structure and higher mobility were considered. Moreover, [21] showed that limited data pilot-aided could have a flexible performance improvement in time-varying channel estimation. A reduced rank autoregressive (AR) has also been used for the channel estimation because of fast time-variant V2X communications channels [22]. In addition, some other research on V2X communication has also received the attention of scholars. The authors of [23] proposed a fuzzy-based channel selection framework for multichannel communications to satisfy location-oriented services in the vehicle cyber-physical system (VCPS). Since V2X communication relies on RSU, physical layer security is potential. Therefore, in [24], the authors propose a secure and energy-efficient virtual multiple-input multiple-output framework that supports simultaneous wireless information and power transmission. This framework can effectively improve the security rate of the Internet of Things system.

To address the difficulty of channel estimation arising from the high mobility in V2X communication, most previous studies exploited the sparse nature of mmWave channels and formulated mmWave channel estimation as a sparse recovery problem [25,26,27]. In [26], a Sparse Bayesian Multi-Task Learning (SB-MTL) is proposed to obtain the dynamic information of the sparse channel. These methods can help achieve a substantial reduction of the training overhead. In order to overcome the grid mismatch issue arising from conventional compressed sensing techniques, super-resolution (off-grid) compressed sensing methods were developed to improve the channel estimation accuracy [28,29]. In [30], an iterative approach for joint estimates of channel impulse response and channel covariance matrix was proposed, while [31] showed that the number of iterations required by [30] may be reduced relying on optimal maximum likelihood sequence detection scheme. The works [32,33] explored the sparse nature of low mobility channels for estimation. Most of those schemes address this problem only by exploiting the sparsity inherent in the channels while ignoring the low-rank characteristics of the received signals. 

In [34], higher-order tensor decomposition techniques were applied to channel estimation for millimeter wave MIMO Orthogonal Frequency Division Multiplexing (OFDM) systems. The authors of [35] developed a Structured CP Decomposition (SCPD) based channel estimation algorithm, which leveraged the fact that the channel can be represented as a low-rank higher-order tensor. It was also adopted to estimate the sparse channel parameters in [36]. The method, however, does not fully exploit multiple RF chains and requires that the channel must be fixed during the estimation process that takes many subframes/frames in the time domain. To further exploit the joint sparse and low-rank structure of mmWave channels. The authors of [37,38,39] have proposed different channel estimation algorithms. However, these schemes can only estimate the quasi-static wireless channel. For V2X communication under high-speed motion, there is currently no evidence that the channel information can be accurately obtained.

In this paper, we propose a sparse Bayes tensor and DOA tracking inspired channel estimation for V2X millimeter wave massive MIMO system. Generally, the scatters around each vehicle are about the same or higher than the vehicle, which means that the signal received by the user’s antennas may arrive from any direction after bounced off the scatters. By exploiting the sparse scattering characteristics of the channel, we transform the channel estimation into a sparse recovery problem. In the process of gridding the transmitting and receiving signals area, the quantization error is inevitably introduced. In order to reduce the influence of such errors, both the receiving and transmitting angle grids should have super-resolution. Furthermore, we take the low-rank characteristics of the received signals into consideration rather than singly using the traditional sparse prior. The sparse Bayes tensor is used to calculate the angle offset. Through exploiting the sparse Bayes tensor, a direction of arrival (DOA) tracking method is developed to acquire the angle value at the next moment, which equals the sum of the angle value at the previous moment and the offset. The proposed approaches are evaluated over the different speeds of the vehicle scenarios and compared to the other methods. Simulation results validated the theoretical analysis and demonstrate that the proposed solution outperforms a number of state-of-the-art researches.

The rest of this paper is structured as follows. The V2X communication system model in the urban road scene is analyzed, and the channel model is established in Section 2. Section 3 presents the channel parameters estimation algorithm. In Section 4, we propose the DOA tracking algorithm based on sparse Bayes tensor. Section 5 shows the simulation results. Finally, this paper is concluded in Section 6.

***Notations*:** We shall denote a scalar by lower-case letters (*a*, *b*, …), column vectors by bold lower-case letters (***a***, ***b***, …), matrices by bold upper-case letters (***A***, ***B***, …), and tensors by upper-case calligraphic letters (A, B, …). For a given matrix ***A***, ***A***^C^, ***A***^T^, ***A***^H^, ***A***^−1^, and ***A***^↑^ denote its conjugate, transpose, conjugate transpose, inverse, pseudo-inverse, respectively. ⊗, ⊙ and ◯ define the Kronecker, Khatri-Rao, and the outer product, respectively. ‖**·**‖_0_, ‖**·**‖_1_, ‖**·**‖_2_ and ‖**·**‖_F_ represents the 0-norm, the first-order norm, the second-order norm of a matrix and Frobenius norm of the matrix, respectively.

## 2. V2X Communication Based on Millimeter Wave Massive MIMO System

### 2.1. Urban Road V2X Communication Scene

In this section, we consider the millimeter wave massive MIMO system with the uniform linear array antenna (ULA) is depicted. For millimeter wave massive MIMO scenario consisting of a Roadside Unit (RSU) and multiple vehicles. With the high speed of the vehicle, the V2X technologies retain the required to have low latency and high reliability in fast-moving environments. To achieve high data rates and stable data transmission for V2X communication, it exists two issues that need to be resolved: (1) accurately estimation of the V2V/V2I channel state information with vehicles under high-speed motion. (2) Efficiently updating of CSI or DOA is extremely needed for the fast time-varying channel. However, a considerable amount of research is for quasi-static channel estimation and failed to update the CSI in time. In this paper, we propose a scheme, which is decoupled into two separated stages, including rapidly initial channel estimation followed by fast DOA tracking.

### 2.2. System Model

Considering the channel estimation of downlink millimeter-wave large-scale MIMO vehicle networking communication system. RSU communicates with vehicles (i.e., V2I communication). RSU is equipped with *N_T_* transmitting antennas and *M_T_* RF chains, while vehicles are equipped with *N_R_* receiving antennas and *M_R_* RF chains. The number of RF chains of RSU or vehicles is limited, usually *M_T_* ≤ *N_T_*, *M_R_* ≤ *N_R_*.

**Figure 1:**
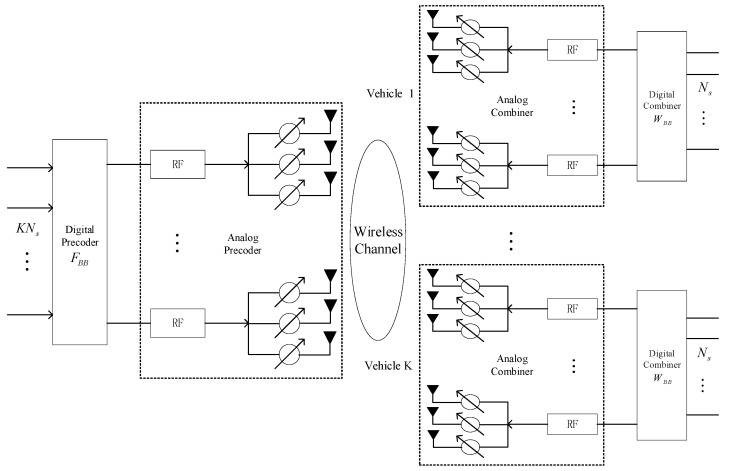
Adopted dynamic hybrid precoding structure.

For each time slot, the RSU uses *M_T_* precoding vectors to precode the transmitted training symbols *s* at consecutive moments, and the vehicle simultaneously uses *M_R_* combination vectors (each combination vector corresponds to a RF chain) to combine and receive signals. Since this work copes with the channel estimation problem, the design of the precoding matrices is not discussed here. We assume that the precoding matrix ***F****_tr_* is known and adopted dynamic hybrid precoding structure in [40] and shown in Figure 1. We assume that the RSU transmits the same training symbol ***s*** in all transmissions. The received signal ***Y***(*t*) ℂ*^M_R_×M_T_^* obtained at the vehicle can be shown as

(1) $$Y(t)=W_{tr}^{H}H\left(t\right)F_{tr}+W_{tr}^{H}N\left(t\right)$$

where ***H***(*t*) ℂ*^N_R_×N_T_^* denotes the mmWave channel at a certain moment from the RSU to the UE, ***W****_tr_* = [***w****_tr_*_,1_,…,***w***_*tr*,*M_R_*_] ℂ*^N_R_×N_T_^* and ***F****_tr_* = [***f****_tr_*_,1_***s***,…,***f****_tr_*_,*M_T_*_***s***] ℂ*^N_T_×M_T_^* denote training combination matrix and precoding matrix respectively, $w_{tr,p}^{H}$ and ***f****_tr_*_,*q*_ denote training combination vector and training precoding vector, respectively. Moreover, $w_{tr,p}^{H}$ satisfy $w_{tr,p}^{H}=\frac{1}{\sqrt{N_{R}}}e^{j\zeta_{i,j}}\left(\zeta_{i,j}\in ℜ\right)$ with set $ℜ=\left\{0,\frac{2\pi }{N_{R}},\frac{2\pi \cdot 2}{N_{R}},\cdots ,\frac{2\pi \left(N_{R}-1\right)}{N_{R}}\right\}$, and the phase *ξ_i,j_* of ***W****_tr_* is selected randomly and uniformly from set R. ***N***(*t*) represents noise matrix.

### 2.3. Channel Model

Because of the high speed of the vehicle, the channel characteristic is time-varying in the time domain and Doppler frequency shift in the frequency domain. Therefore, the Doppler frequency shift is introduced into the channel model and combined with the conventional millimeter wave channel. The time-varying geometric channel model is given by

(2) $$H(t)=\sum_{l=1}^{L}\alpha_{l}(t)a_{R}(\theta_{l}(t))a_{T}(\phi_{l}(t))e^{j2\pi f_{l}T_{s}t}$$

where *L* denotes the number of channel paths. Because millimeter wave channels usually exhibit limited scattering characteristics, *L* is very small. The scalar *α_l_*(*t*)*~*CN(0,1) represents the complex path gain (including path loss), *θ_l_*(*t*) and *ϕ_l_*(*t*), respectively, represent the angle of arrival (AOA) and angle of departure (AOD) of the *l-th* path. *f_l_* and *Ts* represent Doppler shift and sampling period, respectively. ***a_R_***(*θ_l_*(*t*)) ℂ^*N_R_*×1^ and ***a_T_***(*ϕ_l_*(*t*)) ℂ^*N_T_*×1^ represent the array guidance vectors of the receiving and transmitting antennas respectively, which are defined as

(3) $$a_{R}(\theta_{l}(t))={\left[1,\cdots ,e^{j(N_{R}-1)\frac{2\pi }{\lambda }d\sin(\theta_{l})}\right]}^{T}$$

(4) $$a_{T}(\phi_{l}(t))={\left[1,\cdots ,e^{j(N_{T}-1)\frac{2\pi }{\lambda }d\sin(\phi_{l})}\right]}^{T}$$

where *λ* denotes wavelength, and *d* denotes antenna distance, which satisfied *d* = *λ*/2.

We assume that the vehicle moves in a straight line at a uniform speed, and AoAs/AoDs and the complex gain can be considered to change slowly. Therefore, the channel parameters *θ_l_*(*t*), *ϕ_l_*(*t*) and *α_l_*(*t*) have remained constant within a transmission frame by

(5) $$\begin{array}{l} \theta_{l}(t)=\theta_{l}\\ \phi_{{}_{l}}(t)=\phi_{l}\\ \alpha_{l}(t)=\alpha_{l} \end{array}$$

Substituting Equation (5) into Equation (2), we can obtain

(6) $$\begin{array}{ll} H(t) & =\sum_{l=1}^{L}\alpha_{l}a_{R}(\theta_{l})a_{T}(\phi_{l})e^{j2\pi f_{l}T_{s}t}\\ & =\sum_{l=1}^{L}\rho_{l}(t)a_{R}(\theta_{l})a_{T}(\phi_{{}_{l}}) \end{array}$$

where $\rho_{l}(t)=\alpha_{l}e^{j2\pi f_{l}T_{s}t}$ is the gain factor of the fast fading channel, which varies with the Doppler frequency shift.

According to Equation (6), the received signal matrix in Equation (1) can be rewritten as

(7) $$Y(t)=W_{tr}^{H}\sum_{l=1}^{L}\rho_{l}(t)a_{R}(\theta_{l})a_{T}^{H}(\phi_{l})F_{tr}+W_{tr}^{H}N(t)$$

Then, our next work is to estimate the channel parameters in Equation (7). 

## 3. Channel Parameters Estimation

### 3.1. AOAs/AODs Estimation

Generally, the scatters around each vehicle are about the same or higher than the vehicle, which means that the signal received by the user’s antenna may arrive from any direction after bounced off the scatters. However, the RSU is usually deployed higher than the surrounding scatters, thus that the effective path corresponding to a specific vehicle at the RSU is limited to a smaller angle area. Therefore, this article defines the AOAs/AODs areas of the effective propagation path as $\theta_{BW}\subseteq [-\frac{\pi }{2},\frac{\pi }{2}]$ and $\varphi_{BW}\subseteq [-\frac{\pi }{2},\frac{\pi }{2}]$, respectively. Then the AOAs and AODs area are gridded according to the sets Γ*_R_* and Γ*_T_*, respectively. The definitions of Γ*_R_* and Γ*_T_* are shown in Equations (8) and (9).

(8) $$\Gamma_{R}=\left\{\theta_{g_{r}}:\sin(\theta_{g_{r}})=(\sin(\theta_{max})-\sin(\theta_{min}))\frac{g_{r}-1}{G_{R}}+\sin(\theta_{min}),g_{r}=1,\ldots ,G_{R}\right\}$$

(9) $$\Gamma_{T}=\left\{\varphi_{g_{t}}:\sin(\varphi_{g_{t}})=(\sin(\varphi_{max})-\sin(\varphi_{min}))\frac{g_{t}-1}{G_{T}}+\sin(\varphi_{min}),g_{t}=1,\ldots ,G_{T}\right\}$$

where *G_R_* and *G_T_* are the quantitative grid points of AOA and AOD, respectively. In order to reduce the influence of quantization errors, both the receiving and transmitting angle grids should have super-resolution, generally *G_R_* ≥ 2*N_R_*, *G_T_* ≥ 2*N_T_*. Therefore, we define the dictionary matrix of the user side and the base station side as follows

(10) $$A_{U}=[\alpha_{T}(\varphi_{1}),\cdots ,\alpha_{T}(\varphi_{G_{T}})]\in {\mathbb{C}}^{N_{T}\times G_{T}}$$

(11) $$A_{BS}=[\alpha_{R}(\theta_{1}),\cdots ,\alpha_{R}(\theta_{G_{R}})]\in {\mathbb{C}}^{N_{R}\times G_{R}}$$

The received signal matrix of Equation (7) can be written as

(12) $$Y(t)=W_{tr}^{H}A_{BS}H_{grid}A_{U}^{H}F_{tr}+W_{tr}^{H}N(t)$$

where ***H****_grid_* ℂ*^G_R_×G_T_^* represents the channel matrix after lattice.

Performing column vector operations on Equation (12) as

(13) $$\begin{array}{ll} \bar{Y}(t) & ={\left(A_{U}^{H}F_{tr}\right)}^{T}⊗(W_{tr}^{H}A_{BS})vec(H_{grid})+W_{tr}^{H}N(t)\\ & =\Phi P(t)+\xi (t) \end{array}$$

where ***Φ*** = ($A_{U}^{H}$***F**_tr_*)^*T*^ × ($W_{tr}^{H}$***A***_*BS*_) ℂ*^M_R_M_T_×G_R_G_T_^* represents the known measurement matrix, which denoted by ($\phi_{1}^{H}$,…,$\phi_{M_{T}M_{R}}^{H}$)^*H*^. ***P***(*t*) = *vec*(***H****_grid_*) ℂ^*G_R_G_T_*×1^ represents the scattering coefficient vector under the dictionary matrix representation. *ξ*(*t*) = $W_{tr}^{H}$***N***(*t*) represents noise.

Generally, if all AOAs/AODs are located on the grid, the number of non-zero elements of ***P***(*t*) is *L*. The scattering coefficient of the target is the non-zero element of this sparse vector, and the angle information can be determined by the position of the non-zero element in the vector. However, due to the power leakage problem, the actual number of non-zero elements will be greater than *L*. Therefore, we assume that there are *L*_1_ (*L* < *L*_1_ *≤* min{*G_R_*,*G_T_*}) non-zero elements in ***P***(*t*). Then ***P***(*t*) can be regarded as an *L*_1_-sparse vector.

Through the above analysis, we transform the estimation problem of AOAs/AODs into the sparse recovery problem of ***P***(*t*) during single snapshot data, which can be expressed by

(14) $$\begin{array}{l} \underset{\beta (t)}{\min}||\bar{Y}(t)-\Phi P(t)|{|}_{2}\\ s.t.||P(t)|{|}_{0}\leq L_{1} \end{array}$$

To improve the accuracy of AOAs/AODs estimation, we analyze the first *N* snapshot data of a transmission frame. Then in the sparse recovery process, at most *N* × *L*_1_ non-zero elements need to be recovered. By further stacking *N* continuous snapshot data, a latticed sparse channel matrix model is obtained by

(15) $$Y_{N}=\Phi B+E$$

where ***Y****_N_* ℂ*^M_R_M_T_×N^* is known observation matrix, which denoted by {***y***_1_, …, ***y**_N_*}, ***B*** ℂ*^G_R_G_T_×N^* is unknown sparse coefficient matrix, ***E*** ℂ*^M_R_M_T_×N^* is receiving noise matrix. Since AOAs/AODs remain constant during one frame and the sparsity is unchanged, column vectors ${\left\{P_{i}(t)\right\}}_{i=1}^{N}$ in ***B*** are jointly sparse.

Therefore, Equation (15) is a joint sparse recovery problem, expressed as follows

(16) $$\begin{array}{l} \underset{B}{\min}||Y_{N}-\Phi B|{|}_{F}\\ s.t.||B|{|}_{row,0}\leq L_{1} \end{array}$$

where ‖***B***‖*_row_*_,0_ represents the row sparsity of matrix ***B***, defined as

(17) $$||B|{|}_{row,0}=|\underset{i}{\cup }\mathrm{supp}([B{]}_{:,i})|$$

Due to the limited angular resolution of the dictionary matrix, the sparsity of the coefficient matrix ***B*** may be weakened, leading to larger estimation errors. Therefore, we introduce the adaptive iterative update of the AOA/AOD area. Updating the measurement matrix ***Φ*** according to the estimated AOA/AOD in the previous iteration. The proposed adaptive iterative update algorithm for AOAs/AODs is shown in Algorithm 1.

**Algorithm 1** The proposed adaptive iterative update algorithm for AOAs/AODs.Input: measurement matrix ***Φ***, observation matrix ***Y****_N_*, convergence criteria ε, maximum iteration *L_max_*, number of internal cycles J. Output:
$\hat{L},\;\left\{{\hat{\theta }}_{1},\cdots ,{\hat{\theta }}_{\hat{L}}\right\},\;\left\{{\hat{\varphi }}_{1},\cdots ,{\hat{\varphi }}_{\hat{L}}\right\}$
1. Let the residual ***R*** = ***Y****_N_*, support set Ω = Ø, and iteration number *i* = 1 and *j* = 1.2. **Repeat**3.     $\theta_{\min}^{i,1}=\theta_{\min}$, $\theta_{\max}^{i,1}=\theta_{\max}$, $\varphi_{\min}^{i,1}=\varphi_{\min}$, $\varphi_{\max}^{i,1}=\varphi_{\max}$;4.     **for** *j* ∈ [1,J] do5.          $g_{i}={\mathrm{argmax}}_{g_{i}}{\left\|\phi_{i}^{H}R\right\|}_{F},\;{\hat{\theta }}_{i}=\theta_{\left\lceil g_{i}/G_{T}\right\rceil },\;{\hat{\varphi }}_{i}=\varphi_{g_{i}-(\left\lceil g_{i}/G_{T}\right\rceil -1)G_{T}}$;6.          update Ω = Ω∪{*g*_*i*_}, ***R*** = ***R*** − ΩΩ^↑^***Y****_N_*;7.          compute ε^*i*+1^ = ‖***R***‖_F_/‖ΩΩ^↑^***Y****_N_*‖_F_, Δε = |ε^*i*+1^ − ε^*i*^|;8.          *j* = *j* + 1;9.     **end for**10.    update AOAs/AODs area according to Formulas (18)–(21), and then11.    according to Formulas (8)–(11) to redesign the measurement matrix ***Φ***.12.    *i* = *i* + 1;13.**until***i* > *L_max_* and Δε > ε;14.**return**$\hat{L}=i-1,\;\mathrm{AOAs}\;\left\{{\hat{\theta }}_{1},\cdots ,{\hat{\theta }}_{\hat{L}}\right\}\;\mathrm{and}\;\mathrm{AODs}\;\left\{{\hat{\varphi }}_{1},\cdots ,{\hat{\varphi }}_{\hat{L}}\right\}$

In the *i-th* iteration of the outer loop, the goal is to estimate the AOA/AOD of the *i-th* path. In each inner loop, the best index $g_{i}\in \left\{1,2,\cdots ,G_{R}G_{T}\right\}$ is first selected thus that $\phi_{i}^{H}$ is the most relevant to the residual matrix ***R***, and *g_i_* = argmax‖$\phi_{i}^{H}$***R***‖_F_. Then, we can get roughly ${\hat{\theta }}_{i}$ and ${\hat{\varphi }}_{i}$, and the AOAs/AODs area of the *i-th* path is updated by Formulas (18)–(21):

(18) $$\theta_{\min}^{i,j+1}=\arcsin\left(\sin{\hat{\theta }}_{i}+\frac{\sin\theta_{\max}^{i,j}-\sin\theta_{\min}^{i,j}}{2\gamma }\right)$$

(19) $$\theta_{\max}^{i,j+1}=\arcsin\left(\sin{\hat{\theta }}_{i}-\frac{\sin\theta_{\max}^{i,j}-\sin\theta_{\min}^{i,j}}{2\gamma }\right)$$

(20) $$\varphi_{\min}^{i,j+1}=\arcsin\left(\sin{\hat{\varphi }}_{i}+\frac{\sin\varphi_{\max}^{i,j}-\sin\varphi_{\min}^{i,j}}{2\gamma }\right)$$

(21) $$\varphi_{\max}^{i,j+1}=\arcsin\left(\sin{\hat{\varphi }}_{i}-\frac{\sin\varphi_{\max}^{i,j}-\sin\varphi_{\min}^{i,j}}{2\gamma }\right)$$

where *γ* is the step size.

Based on the updated AOA/AOD area, we redesign the measurement matrix to increase the resolution of the redundant dictionary. Then in each iteration, the support set **Ω** is updated by taking the union of {*g_i_*} and **Ω**, and a new residual is calculated. Then updates the angle quantification sets Γ_R_ and Γ_T_, updating ${\hat{A}}_{U}=[\alpha_{T}(\varphi_{1}),\cdots ,\alpha_{T}(\varphi_{\hat{L}})]$,

$${\hat{A}}_{BS}=[\alpha_{R}(\theta_{1}),\cdots ,\alpha_{R}(\theta_{\hat{L}})]$$

This algorithm has the following advantages: (1) the adaptive update process reduces the power leakage generated by the quantization grid and improves the accuracy of AOA/AOD estimation. (2) Since the position of the non-zero element in ***B*** is not related to the Doppler frequency shift, the algorithm is robust to the Doppler frequency shifts.

### 3.2. Fast Fading Channel Gain Estimation

In this stage, the path gain and Doppler shift will be obtained based on the AOAs/AODs estimated at the first stage. However, traditional methods based on compressed sensing retain failed to estimate the channel efficiently. As a mathematical tool that can quickly process large amounts of data, tensor decomposition theory is used to estimate channel gains and Doppler shift.

It uses the downlink transmission frame structure, which consists of a channel estimation phase (T_1_ time slots) and multiple subframes. Each subframe includes a fast DOA updating phase (T_2_ time slots) and a data transmission phase (T_3_ time slots).

Alternatively, we can rewrite (7) as the outer product of the rank-one matrix. The AOAs/AODs are given by

(22) $$\begin{array}{l} {\tilde{a}}_{R}(\theta_{l})=W_{tr}^{H}a_{R}(\theta_{l})\\ {\tilde{a}}_{T}(\varphi_{l})=F_{tr}^{T}a_{T}^{C}(\varphi_{l}) \end{array}$$

where ${\tilde{a}}_{R}(\theta_{l})\in {\mathbb{C}}^{M_{R}\times 1}$ and ${\tilde{a}}_{T}(\varphi_{l})\in {\mathbb{C}}^{M_{T}\times 1}$ represent transmitter matrix and receiver matrix, respectively. 

Therefore, received signals can be expressed as

(23) $$Y(t)=\sum_{l=1}^{L}\rho_{l}(t){\tilde{a}}_{R}(\theta_{l})\circ {\tilde{a}}_{T}(\varphi_{l})+W_{tr}^{H}N(t)$$

Assuming that in T continuous time slots, the received signal is expressed as a tensor by

(24) $$Y=\sum_{l=1}^{L}{\tilde{a}}_{R}(\theta_{l})\circ {\tilde{a}}_{T}(\varphi_{l})\circ {\tilde{\rho }}_{l}+N$$

where Y ℂ*^M_R_×M_T_×T^* represents receiving signal tensor, ${\tilde{\rho }}_{l}\in {\mathbb{C}}^{T\times 1}$ is a column vector consisting of fast fading channel gains, i.e., ${\tilde{\rho }}_{l}={\left[\rho_{l}(1),\cdots ,\rho_{l}(T)\right]}^{T}$, and N ℂ*^M_R_×M_T_×T^* represents the noise tensor.

According to the properties of tensor decomposition, the received signal can be expressed as the n-mode product of low-rank tensors by extending the time dimension. Referring to the typical CANDECAMP/PARAFAC (CP) decomposition, we can get

(25) $$Y=I\times_{1}D_{1}\times_{2}D_{2}\times_{3}D_{3}+N$$

where I ℂ*^L×L×L^* is identity core tensor. The factor matrix ***D***_1_ ℂ*^M_R_×L^*, ***D***_2_ ℂ*^M_T_×L^*, and ***D***_3_ ℂ*^T×L^* are defined as in Equations (26)–(28).

(26) $$D_{1}=[{\tilde{a}}_{R}(\theta_{1}),\cdots ,{\tilde{a}}_{R}(\theta_{L})]$$

(27) $$D_{2}=[{\tilde{a}}_{T}(\varphi_{1}),\cdots ,{\tilde{a}}_{T}(\varphi_{L})]$$

(28) $$D_{3}=[{\tilde{\rho }}_{1},\cdots ,{\tilde{\rho }}_{L}]$$

In order to obtain the three factor matrices ***D***_1_, ***D***_2_, and ***D***_3_, the traditional algorithm based on PARAFAC decomposition (CP decomposition) adopts an alternate least square method. However, these algorithms require careful design of precoding and combinatorial matrices to ensure the uniqueness of the CP decomposition, and are suitable for static mmWave channel estimation. To achieve rapid channel estimation in the V2X communication environment, we propose a new algorithm, which will be described in detail in the next.

Similarly, stacking the received signal matrix of T_1_ consecutive time slots, we can obtain the following received signal tensor

(29) $$Y^{\left(T_{1}\right)}=I^{}\times_{1}D_{1}^{}\times_{2}D_{2}^{}\times_{3}D_{3}^{}+N^{\prime }$$

where Y ^(T^^1)^ is the received signal tensor in the fast-fading channel gain estimation stage, $N^{\prime }$ is the corresponding noise tensor, and the factor matrix ***D***_3_ is defined as

(30) $$D_{3}=[\rho_{1},\cdots ,\rho_{L}]\in {\mathbb{C}}^{T_{1}\times L}$$

(31) $$\rho_{l}={\left[\rho_{l}(1),\cdots ,\rho_{l}(T_{2})\right]}^{T}\in {\mathbb{C}}^{T_{1}\times 1}$$

Considering that no matter what the uniqueness condition of CP decomposition is, for the received signal tensor Y ^(T^^1)^ of Equation (29), the following equation always holds

(32) $$Y_{\;(3)}^{\left(T_{1}\right)}=D_{3}^{}{\left(D_{2}^{}⊙D_{1}^{}\right)}^{T}$$

For Equation (32), we can construct factor matrices ${\hat{D}}_{1}^{}$ and ${\hat{D}}_{2}^{}$ from the previously estimated AOAs/AODs. Therefore, the factor matrix ${\hat{D}}_{3}^{}$ can be estimated by

(33) $${\hat{D}}_{3}^{}=Y_{\;(3)}^{\left(T_{1}\right)}{\left[{\left({\hat{D}}_{2}^{}⊙{\hat{D}}_{1}^{}\right)}^{T}\right]}^{↑}$$

Then, we adopt a correlation-based scheme to estimate the Doppler frequency shift, which is given by

(34) $${\hat{f}}_{l}=\underset{f_{i}}{\arg\max}\frac{\left|{\left[{\hat{D}}_{3}^{}\right]}_{:,l}^{H}\rho (f_{i})\right|}{{\left\|{\left[{\hat{D}}_{3}^{}\right]}_{:,l}^{}\right\|}_{2}{\left\|\rho (f_{i})\right\|}_{2}},1\leq i\leq I,{\hat{f}}_{i}\in \left[0,f_{\max}\right]$$

(35) $$f_{\max}=\frac{f_{c}v}{c}$$

where $f_{\max}$ is the maximum Doppler shift, $f_{c}$ is the carrier frequency, v is the vehicle speed, and *c* is the speed of light. This correlation scheme involves one-dimensional optimization. First, use a coarse grid to segment the target area, and then gradually refine the search around the possible grid points to perform effectively.

Next, we estimate the path gain, which can be expressed by

(36) $${\hat{\alpha }}_{l}={\left[\rho (f_{l})\right]}^{↑}{\left[{\hat{D}}_{3}^{}\right]}_{:,l}$$

(37) $$\hat{\alpha }=({\hat{\alpha }}_{1},\cdots ,{\hat{\alpha }}_{\hat{L}})$$

Finally, the channel matrix is restored based on the estimated AOAs/AODs and path gain by

(38) $$\hat{H}={\hat{A}}_{BS}diag(\hat{\alpha }){\hat{A}}_{U}^{H}$$

where $diag(\hat{\alpha })$ represents the diagonalization operation, the element of the path gain vector $\hat{\alpha }$ is taken as the main diagonal element, and the other elements keep zero.

## 4. Fast DOA Tracking Algorithm

DOA tracking, also known as angle of arrival tracking, refers to the real-time estimation of DOA of moving targets. In a real scene, the fast movement of vehicles leads to the rapid time-varying of the wireless channel, which makes it difficult to accurately acquire the CSI. To solve this problem, we proposed a DOA tracking method based on a sparse Bayes tensor. Specifically, based on the previously estimated angle, the sparse Bayes tensor is used to calculate the angle offset. The angle value at the next moment can be acquired, which equals the sum of the angle value at the previous moment and the offset.

We first perform windowing processing on the sub-frame data, the window function is a rectangular window, and the length is *U*(*U* > 1). We assume that at the *n-th* moment, the DOA of the multipath signal can be expressed as ***θ***(***n***) = [***θ***_1_(***n***),…, ***θ****_L_*(***n***)]^*T*^. The steering vector at the next moment can be estimated according to Taylor expansion by

(39) a(θ(n+1))≈a(θ(n))+c(θ(n))(θ(n+1)−θ(n)) (1<n≤U)

where ***c***(***θ***(*n*)) represents the offset of the steering vector from *n-th* moment to (*n* + 1)*-th* moment. Then we let ***η***(***n* + 1**) be the offset of DOA, which is expressed as

(40) η(n+1)=θ(n+1)−θ(n)

We assume that

(41) $$Z(n)=[a(\theta_{1}(n)),\cdots ,a(\theta_{L}(n))]$$

(42) $$C(n)=[c(\theta_{1}(n)),\cdots ,c(\theta_{L}(n))]$$

(43) $$\eta (n)={\left[\eta_{1}(n),\cdots ,\eta_{L}(n)\right]}^{T}$$

According to Equation (39), the matrix form of the steering vector is given by

(44) Z(n)=Z(n−1)+C(n−1)diag(η(n))

Then, according to the estimated ${\hat{D}}_{1}$, ${\hat{D}}_{2}$, and ${\hat{D}}_{3}$, we can obtain the received signals as

(45) $$\hat{Y}=I\times_{1}{\hat{D}}_{1}\times_{2}{\hat{D}}_{2}\times_{3}{\hat{D}}_{3}$$

The 1-mode unfolding matrices of Equation (45) admit the following factorizations in terms of the factor matrices

(46) $${\hat{Y}}_{\left(1\right)}={\hat{D}}_{1}{\left({\hat{D}}_{2}⊙{\hat{D}}_{3}\right)}^{T}$$

Next, we need to get the angular offset ***η***(*n*). However, in Equation (46), ${\hat{D}}_{1}$ includes the AOA estimated in the previous stage. The Formula (44) reflects the AOA update process at two adjacent moments. In fact, the initial AOA information contained in ${\hat{D}}_{1}$ is exactly what the DOA tracking algorithm needs to be updated. Therefore, based on the known ${\hat{Y}}_{\left(1\right)}$, ${\hat{D}}_{2}$, and ${\hat{D}}_{3}$, we substitute ***Z***(***n***) instead of ${\hat{D}}_{1}$ into Equation (46) to construct a DOA tracking model. Then we let $S(n)={\left({\hat{D}}_{2}⊙{\hat{D}}_{3}\right)}^{T}$ and a new observation model can be expressed by

(47) $$Y_{\left(1\right)}(n)=(Z(n-1)+C(n-1)diag(\eta (n)))S(n)+N(n)$$

where ***N***(***n***) represents the noise at the *n-th* moment.

Thus far, we have deduced offset expression of DOA. In the following tracking process, we need to continuously update the value of ***η***(*n*) through iteration. The initial values of ***Z***(***n***) and ***S***(***n***) are given by the previous AOAs/AODs estimation process. Subsequently, the values of ***Z***(***n***) and ***S***(***n***) are obtained according to Equations (44) and (47), respectively. Thus, Equation (47) can be transformed into a minimum *l*_1_ constraint problem, which can be expressed as

(48) $$\tilde{S}(n)=\arg\min\left\{{\left\|Y_{\left(1\right)}(n)-Z(n)S(n)\right\|}_{2}^{2}+\chi {\left\|S(n)\right\|}_{1}\right\}$$

where ***χ*** is a normalization factor. After getting the initial value of ***Z***(***n***) and ***S***(***n***), we can get ***η***(*n*) by minimizing the following function:

(49) $$\begin{array}{ll} \tilde{\eta }(n) & =\arg\min E\left\{{\left\|Y_{\left(1\right)}(n)-[Z(n-1)+C(n-1)diag(\eta (n))]\tilde{S}(n)\right\|}_{2}^{2}\right\}\\ & =\arg\min\left\{\eta^{T}(n)J\eta (n)-2K^{T}\eta (n)+V\right\} \end{array}$$

(50) $$J=Re\left\{\bar{C^{H}C}(\mu \mu^{H}+\Sigma )\right\}$$

(51) $$K=Re\left\{\frac{1}{L}diag(\mu C^{H}(Y_{\left(1\right)}(n)-Z\mu ))\right\}-Re\left\{diag(C^{H}Z\Sigma )\right\}$$

where *V* is a constant, ***J*** and ***K*** represent parameter matrix, ***μ*** and Σ are the mean and variance of ***S***(***n***). Therefore, the minimum constraint problem can be simplified as

(52) $$\tilde{\eta }(n)=2K^{T}J^{-1}$$

Assuming that the signals in the data are independent of each other, and ***N***(***n***) obeys a complex Gaussian distribution, the mean is 0 and the variance is $\delta_{n}^{2}$. Then, we can get the mean and variance of ***S***(***n***) by [41]:

(53) $$\mu =\delta_{n}^{-2}\Sigma Z^{H}(n)Y_{\left(1\right)}(n)$$

(54) $$\Sigma =(\delta_{n}^{-2}Z^{H}(n)Z(n)+\Lambda {)}^{-1}$$

where $\Lambda =diag(\alpha_{1},\cdots ,\alpha_{L})$ and $\{\alpha_{i}\}\left(i=1,\cdots ,L\right)$ are hyperparameters, which can be estimated by performing a type-II maximum-likelihood (or evidence maximization) procedure [42]. Using Equation (52), we can estimate the angle offset $\tilde{\eta }(n)$.

## 5. Numerical Results

This section considers the deployment scenarios of 5G V2I systems in urban highways. In order to verify the channel estimation algorithm proposed in this article, we need to specify the simulation parameters. Here, we stipulate a mmWave MIMO-OFDM system operates in the 73 GHz frequency band with a bandwidth of 20 MHz and the number of FFT points N = 64. A frequency separation Δ*f* = 1/Ts = 0.3125 MHz, such that the symbol period Ts = 3.2 μs. The length of the cyclic prefix was set to 4 μs, with 4 subcarriers used as pilots, and the 48 subcarriers remaining were used for data transmission. We considered a V2I system consisting of one BS. The BS has *M*_*T*_ = 4 RF chains, and the vehicle had a single RF chain. For channel model in Equation (7), we took dictionary matrix grid size *G*_*T*_ = 2*N*_*T*_, *G*_*R*_ = 2*N*_*R*_. The AOAs and AODs were assumed to take continuous values and uniformly distributed in *θ_BW_* = [−π/2, π/2] and *φ_BW_* = [−π/2, π/2], respectively. Other parameters included, *ε*_0_ = 10^−2^, γ = 2, and *L*_*max*_ = 5. Here, *J* = 100 was chosen to achieve better compromise between performance and computational complexity. For the transmission frame structure, we set *T*_1_ = 20, *T*_2_ = 5, and *T*_3_ = 45.

In this section, the performance of the proposed channel estimation algorithms was compared with that of the OMP-MTC, AAE, MT-SBL, and SCPD channel estimation algorithms. The normalized mean square error (NMSE) was used to evaluate the estimation accuracy, which is defined as

(55) $$NMSE(dB)=10\log_{10}\left(E\left[\frac{{\left\|H-\hat{H}\right\|}_{F}^{2}}{{\left\|H\right\|}_{F}^{2}}\right]\right)$$

where $\hat{H}$ denotes the estimated value of the entire channel ***H***.

**Figure 2:**
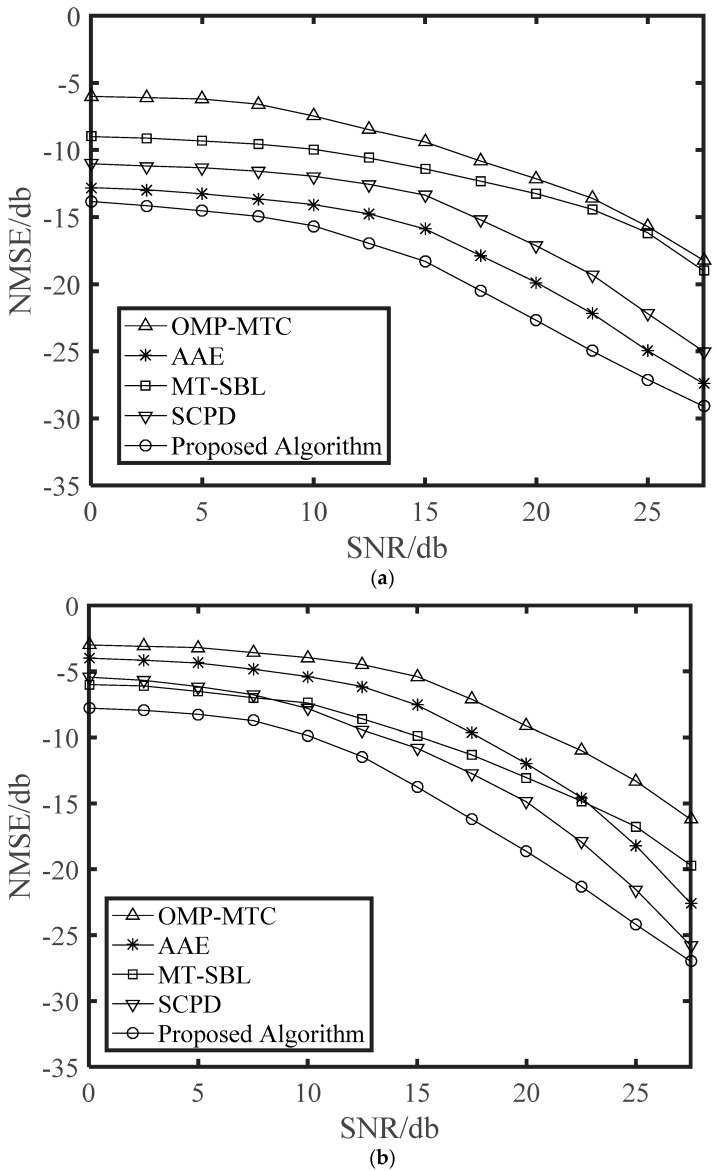
NMSE performance against SNR with a single vehicle, *G*_*T*_ = 128, *G*_*R*_ = 32, *N*_*R*_ = 16, and *N*_*T*_ = 64 antennas. (**a**) *v* = 120 km/h. (**b**) *v* = 200 km/h.

Figure 2 shows the NMSE performance versus SNR with *v* = 120 km/h and *v* = 200 km/h. It can be seen from Figure 2 that the OMP-MTC estimators performed poorly for all the SNR range since it was designed for static channels. For the highway environment, the Doppler frequency shift caused by the high-speed movement of the vehicle will have a great impact on the algorithm. As expected, the AAE algorithm obtained a good performance, while it still had some performance loss compared with our proposed algorithm. The reason for that was the fact that at such speed, variations of AOAs/AODs were much faster than that of path gains resulting in inaccurate estimation. The MT-SBL and SCPD estimators performed poorly due to the fact that they were singly developed channel sparsity characteristics or low-rank characteristics. Overall, our proposed algorithm had better performance than other algorithms. The above phenomenon may be due to the joint sparse, and low-rank characteristic was utilized in our proposed algorithm.

**Figure 3:**
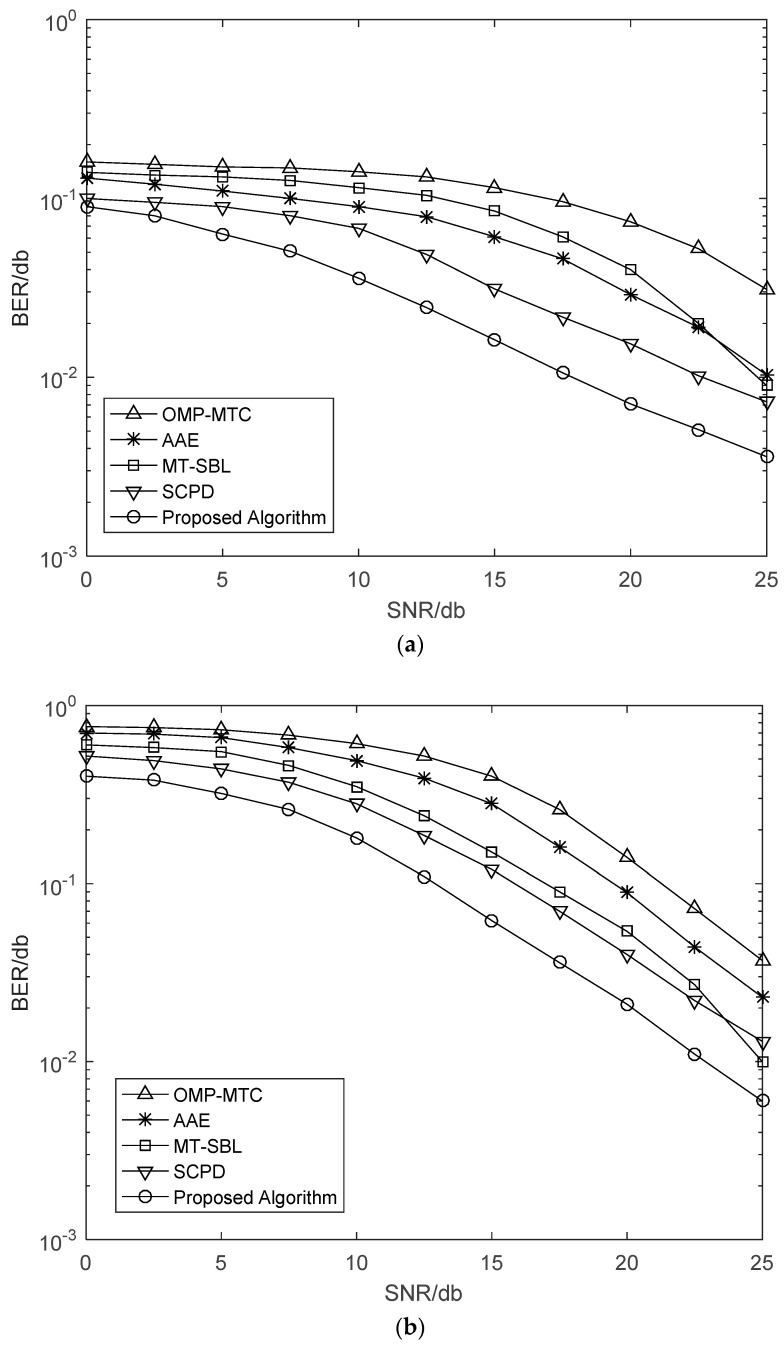
BER performance against SNR with a single vehicle, *G*_*T*_ = 128, *G*_*R*_ = 32, *N*_*R*_ = 16, and *N*_*T*_ = 64 antennas. (**a**) *v* = 120 km/h. (**b**) *v* = 200 km/h.

Figure 3 further illustrates the bit error rate (BER) curves of different vehicle speed *v* = 120 km/h and *v* = 200 km/h. We can observe that the SCPD and proposed algorithm have the approximative BER performance, and both of them outperform other estimation algorithms, especially in the high SNR regime. Moreover, there exists a performance gap between the AAE and OMP-MTC because the number of channel measurements was not sufficient for the OMP-MTC in the relatively higher speed. Meanwhile, we can observe that the MT-SBL has slightly better NMSE performance than that of the OMP-MTC. However, both of them have performance gaps compared with the proposed algorithm since they cannot fully exploit the diversity gain from structural low-rank and sparsity.

**Figure 4:**
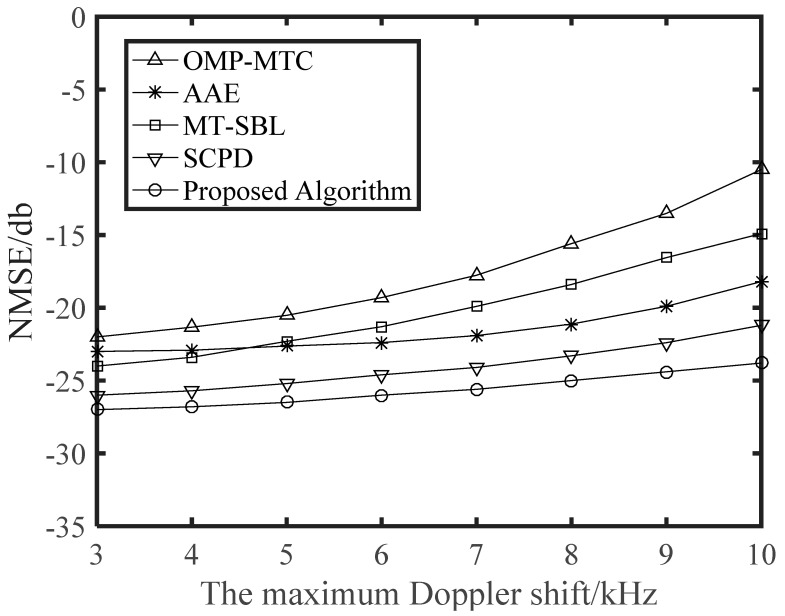
NMSE performance against the maximum Doppler shift with *G*_*T*_ = 128, *G*_*R*_ = 32, SNR = 20 dB, *N*_*R*_ = 16, and *N*_*T*_ = 64 antennas. The number of vehicles is 4, and the distance between vehicles is greater than 50 m.

**Figure 5:**
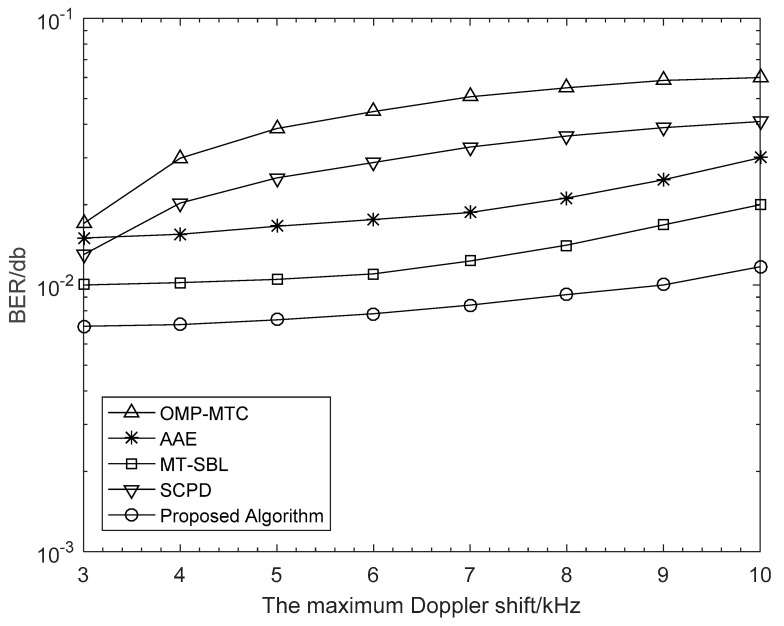
BER performance against the maximum Doppler shift with *G*_*T*_ = 128, *G*_*R*_ = 32, SNR = 20 dB, *N*_*R*_ = 16, and *N*_*T*_ = 64 antennas. The number of vehicles is 4, and the distance between vehicles is greater than 50 m.

To show how Doppler shift influences the system performance, we depicted the NMSE and BER curves against the maximum Doppler shift *f_max_* in Figure 4 and Figure 5, respectively, for a range of 3 kHz to 10 kHz, with the corresponding velocity range of 44.4 km/h to 148.0 km/h. Obviously in Figure 4, the NMSE curves monotonically increased with *f_max_*, which was mainly as the result of that the approximation error resulting from the linear interpolation becomes more severe as the Doppler shift increases. Then, in Figure 5, we examined the BER performance of different algorithms as a function of the maximum Doppler shift. Visibly, the other algorithms lacked robustness when the maximum Doppler shift was very high. It is vital to point out that, compared with other algorithms, our proposed scheme maintained a stable and better estimation performance with the increase of Doppler shift. This is a benefit from that our proposed algorithm captures the changes of DOA information in the time-varying channels.

**Figure 6:**
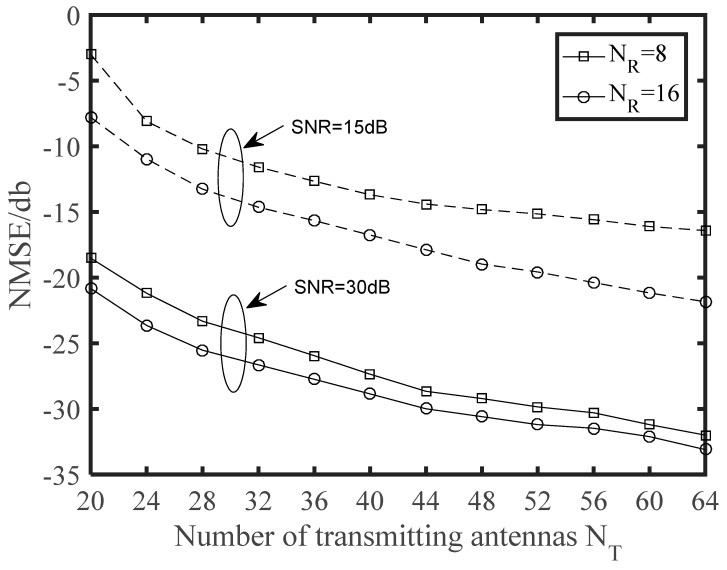
NMSE performance against the number of the transmitting antennas *N_T_*. The number of vehicles is 1, and its speed is *v* = 120 km/h.

**Figure 7:**
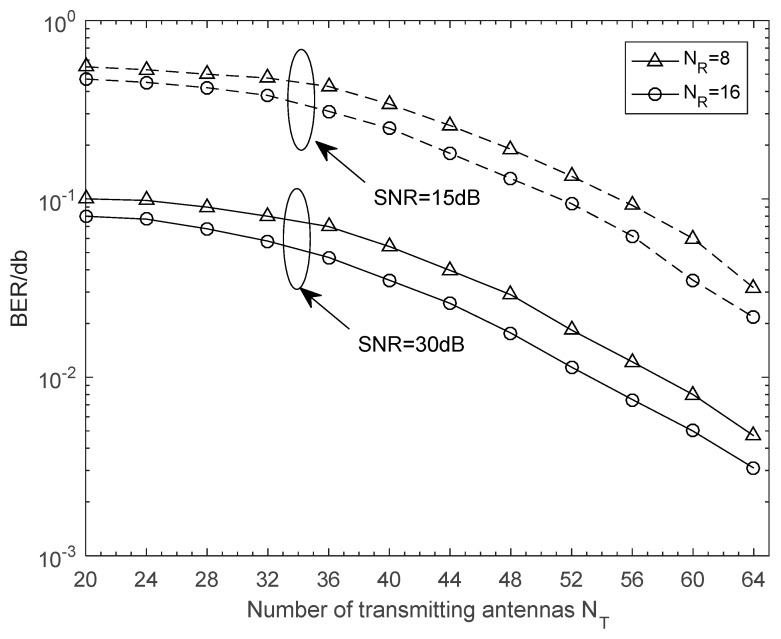
BER performance against the number of the transmitting antennas *N_T_*. The number of vehicles is 1, and its speed is *v* = 120 km/h.

Finally, we depict NMSE and BER curves of the proposed time-varying channel estimation scheme when the number of transmitting antennas was changing. The NMSE performance versus the number of transmitting antennas is illustrated in Figure 6. The schemes were simulated for the cases with different receiving antennas in different SNR. It is clear that NMSE curves monotonously decreased with *N_T_*. When SNR = 30 was considered, the *N_R_* = 8 scheme approached the corresponding *N_R_* = 16 scheme, and thus a greater number of receiving antennas was not generally required in practical application. From Figure 7, we can see that the BER performance was improved with increasing numbers of antennas. This is a benefit from that more antennas at the BS or the vehicle can obtain larger beamforming gains for higher accuracy in path gains estimation. Note that a great deal of antennas will also complicate the V2X system and increase the energy consumption of the vehicle. From Figure 2, Figure 3, Figure 4, Figure 5, Figure 6 and Figure 7, we can intuitively see that the proposed algorithm was effective for time varying channel parameters estimation and channel tracking under different vehicle environments.

## 6. Conclusions

With this paper, we proposed a channel estimation scheme for V2X millimeter wave massive MIMO system. By exploiting the sparse scattering characteristics of the channel, we transformed the channel estimation into a sparse recovery problem. A gridding scheme with super-resolution is adopted to complete the channel state acquisition. At the same time, in order to further reduce the quantization error, the redundant dictionary matrix is also designed to have super-resolution. Furthermore, by utilizing joint sparse and low-rank characteristics, a DOA tracking method is proposed to provide a significant angle information update for tracking fast time-varying vehicular channels. Simulation results confirmed that the proposed method is effective in estimating the fast time-varying vehicular channels. Moreover, the proposed estimation schemes and algorithms in this paper can also be easily extended to other V2X systems with a slight modification.

## Data Availability

Data sharing not applicable.
